# Supplementary material for: Single Cell ADNP Predictive of Human Muscle Disorders: Mouse Knockdown Results in Muscle Wasting
Source: Cells. 2020 Oct 19;9(10):2320. doi: 10.3390/cells9102320 (PMC7603382; doi:10.3390/cells9102320)
Supplement: Supplementary file 1 [file cells-09-02320-s001.pdf]

Table S1. Selected muscle diseases.

Disease	Disease description
**Myotonic dystrophy (DM)**	The most common adult muscular dystrophy, is characterized by autosomal dominant progressive myopathy. Two distinct forms caused by similar mutations in two different genes have been identified: myotonic dystrophy type 1 (DM1, with mutations in myotonin-protein kinase) and myotonic dystrophy type 2 (DM2 with mutations in the cellular nucleic acid-binding protein gene, *CNBP*).
**Duchenne muscular dystrophy (DMD)**	An X-linked recessive disorder in the dystrophin gene (1/5000 boys) presents in early childhood (age of 4) with proximal muscle weakness. Dystrophin function is linked with microtubule binding. The absence of dystrophin causes DMD.
**Becker muscular dystrophy (BMD)**	Mutations resulting in a reduced amount or shortened dystrophin protein cause BMD. BMD has a mean onset at 12 years, with loss of ambulation occurring around the third decade of life, and a life expectancy that is similar to the general population, contrasting DMD.
**Pompe disease**	A rare and deadly muscle disorder, combines a glycogen storage disorder, a lysosomal disorder, and an autophagic myopathy, caused by mutations in the gene coding for acid alpha-glucosidase (GAA, breaking down glycogen in the lysosome acidic milieu).
**Tibial muscular dystrophy (TMD)**	A late onset, autosomal dominant distal myopathy, results from mutations in the two last domains of titin (associated with the unfolded protein response and altered autophagy).
**Dysferlinopathies**	Harboring dysferlin mutations (*DYSF*). Dysferlin is responsible for membrane resealing, and mutations may result in a defect in membrane repair following mechanical or chemical stress, causing an influx of Ca^{2+} and affecting mitochondrial function.
**Secondary dystroglycanopathies**	Mutations in the fukutin related protein gene (*FKRP*) cause secondary dystroglycanopathies, muscular dystrophies that result from mutations in genes that participate in dystroglycan glycosylation, which is in turn essential for the muscle fibers to adhere to the muscle extracellular matrix.
**Amyotrophic lateral sclerosis (ALS)**	ALS patients also suffer from muscle wasting and our previous studies have shown brain tauopathy in the SOD1-G93A mouse model of ALS, which was corrected by NAP. (See main text, discussion).

Table S2. A tabular summary of the different patients and aged matched controls characterizations.

Disease	Pompe	DMD	DM2	TMD	BMD	DYSF	FKRP	ALS
**Age of sampling**	**Control:** 2m-2y 11 m **Patient:** 1 y 5 m	**Control:** 5-7 y **Patient:** 1-10 y	**Control:** 45-54 y **Patient:** 37-55 y	**Control:** 76-80 y **Patient:** 48-65 y	**Control:** N/A **Patient:** 10-32 y	**Control:** N/A **Patient:** 16-38 y	**Control:** N/A **Patient:** 12-55 y	**Control:** N/A **Patient:** 45-55 y
**Muscle type**	Biceps	Quadriceps	Vastus lateralis	Distal muscles	Typically, from the vastus lateralis	Typically, from the vastus lateralis	Typically, from the vastus lateralis	Typically, from the vastus lateralis
**Sex**	Males & Females	Males	Males & Females	Males	Males	Males & Females	Males & Females	Males

Table S3. G68 off targets. The vast majority have 4 mismatches with the G68 sequence, some have 3 and only 1-off target has 2 mismatches (intronic), making the effects of these targets mostly negligible.

offtargetSeq	mismatchPos	mismatchCount	mitOfftargetScore	cfdOfftargetScore	chrom	start	end	Strand	locusDesc
GAGGGAAGAAGTAGGACTGTGGG	**....*..*..........	4	0.89998622	0.725274725	chr11	1.2E+08	1.2E+08	-	intron:Aspscr1
ATGGGTAGATGCAGAACTGTGGG	.....**....*..*.....	4	0.077952761	0.636886333	chr8	6.1E+07	6.1E+07	-	intron:2700029M09Rik
CTGGGAGGATACAAGACTGTGGG	*.........**.*......	4	0.061157663	0.609022556	chr9	1E+08	1E+08	+	intergenic:Gm5161-Ephb1
ACAGGAAGATGTAAGACTGTTGG	.**...*......*......	4	0.150830932	0.482142857	chr7	1.3E+08	1.3E+08	+	exon:D430042O09Rik
AGGAGTGGAAGTAGGACTGTTGG	.*.*.*...*..........	4	0.760552802	0.462857143	chr9	2.6E+07	2.6E+07	-	intergenic:Gm26190-B3gat1
ATGGGGAGATGCAGGACTTTAGG	.....**....*......*.	4	0.096262749	0.45112782	chr8	1E+08	1E+08	-	intergenic:Bean1-Tk2
AAGGGAGGATGAGGAACTGTGGG	.*.........**.*.....	4	0.07670389	0.415502656	chr3	2.8E+07	2.8E+07	-	intergenic:Tmem212-Pld1
ATTGGAAGATAAAGGACTGTGGG	..*...*...**........	4	0.263094174	0.4	chr19	2.2E+07	2.2E+07	+	intergenic:Gda-Gm3443
ACGGGGAGATGTGGGACTGTGGG	.*...**.....*.......	4	0.228792914	0.399290151	chr16	3.7E+07	3.7E+07	-	intron:Golgb1
GGGGGAGGAGGTAGAACTGTGGG	**.......*....*.....	4	0.371023101	0.395294118	chr11	2.2E+07	2.2E+07	-	intergenic:Gm12047-Wdpcp
AGGGAAGGATGCAGGACTGAAGG	.*..*......*.......*	4	0.343144014	0.387947369	chr15	5.5E+07	5.5E+07	-	intron:Mal2
ATGAGAGTATATAGGACTTTCGG	...*...*..*.......*.	4	0.27680625	0.375	chr7	7.4E+07	7.4E+07	+	intergenic:RP23-32A8.1-Gm26176
GGGGGAGGGTGTAGGACTTTTGG	**......*.........*.	4	0.321904489	0.373333334	chrX	1.1E+08	1.1E+08	-	intergenic:Gm6377/Sh3bgrl-Gm14868
ATGGGAGAATAAAGGAATGTTGG	.......*..**....*...	4	0.150409179	0.373333334	chr16	1.8E+07	1.8E+07	-	intergenic:Vpreb2-Dgcr6
AGAGGGGGATGAAGGACTGTAGG	.**..*.....*........	4	0.41990681	0.36	chr18	7E+07	7E+07	+	intergenic:Rab27b-Dynap
CTGGGAGGGTGTAGAACTTTTGG	*.......*.....*...*.	4	0.086270403	0.358543418	chr7	7.4E+07	7.4E+07	-	intergenic:Slco3a1-Gm7580

TTGGGAGGCTGTGGAACTGTAGG	*.......*...*.*.....	4	0.095256253	0.350748995	chrX	1.7E+08	1.7E+08	+	intergenic:Gm15238-Frmpd4
ATGGAAGAATCTAGAACTGTTGG	....*..*..*...*.....	4	0.212805723	0.349579833	chr16	8.4E+07	8.4E+07	+	intergenic:Rpl21-ps5-Gm24508
ATGGAGGGAAGTGGGACTGTAGG	....**...*..*.......	4	0.294333934	0.346051464	chr1	4.2E+07	4.2E+07	-	intergenic:Gm5973-Gm9915
AAGGGACCATGTAGAACTGTAGG	.*....**......*.....	4	0.275145253	0.336930038	chr6	1E+08	1E+08	-	intergenic:Gm24248-Rybp
ATGAGAGGATAAAGGAATGTTGG	...*......**....*...	4	0.158024834	0.336	chr13	1.2E+07	1.2E+07	+	intergenic:Gm25496-Ryr2
TGGGGAGGAGGAAGGACTGTGGG	**.......*.*........	4	0.648306325	0.336	chr7	5.9E+07	5.9E+07	+	intergenic:Gm26288-Atp10a
ATGGGAGGGTGAAGAACTTTCGG	........*..*..*...*.	4	0.036308406	0.334640523	chr2	6.9E+07	6.9E+07	-	intron:Lrp2
AAGGGAGGCAGAAGGACTGTGGG	.*......**.*........	4	0.396115165	0.33155416	chr3	2.6E+07	2.6E+07	+	intergenic:Gm24250-Nlgn1
GTGGAAGGCTGTAGGACTTTGGG	*...*...*.........*.	4	0.321904489	0.33015873	chr7	3.8E+07	3.8E+07	+	intergenic:Uri1-Ccne1
ATGGGAGAAGATGGGACTGTTGG	.......*.**.*.......	4	0.258140296	0.326086957	chr10	8.3E+07	8.3E+07	+	intergenic:Chst11-Slc41a2
ACAGGAGCATGAAGGACTGTGGG	.**....*...*........	4	0.694060843	0.316483516	chr11	5.2E+07	5.2E+07	+	intron:Sec24a
ATGGGAATATAGAGGACTGTGGG	......**..**........	4	0.243372227	0.3125	chr3	1.3E+08	1.3E+08	-	intergenic:Gm23011-Gm26691
ATGAAAGGAGGAAGGACTGTGGG	...**....*.*........	4	0.618499138	0.312	chr11	5.9E+07	5.9E+07	+	intron:Fam183b
AAGGGATGAAGTAAGACTGTGGG	.*....*..*...*......	4	0.140887716	0.310832025	chr4	1.1E+08	1.1E+08	-	intron:Slc1a7
AGGGAAGGAGGAAGGACTGTAGG	.*..*....*.*........	4	0.648306325	0.2912	chr15	4.3E+07	4.3E+07	+	intergenic:Gm17473-Rspo2
ATGAAAGGATGTGGGACTGATGG	...**.......*......*	4	0.25551675	0.286141304	chr12	3.3E+07	3.3E+07	-	intron:Gm11052
ATGGATGGATGAAGGACTGAAGG	....**.....*.......*	4	0.196530015	0.278571429	chr8	9.5E+07	9.5E+07	-	exon:Cngb1
ACAGGAAGATCTAGGACTGTAGG	.**...*...*.........	4	0.534744255	0.275510204	chr19	4.6E+07	4.6E+07	-	intron:Gbf1

AGGGAAGGATGAAGGATTGTAGG	.*..*......*....*...	4	0.299915	0.271786667	chr5	1.1E+08	1.1E+08	-	intergenic:E130006D01Rik-Miat/MIAT_exon5_3/MIAT_exon5_2/Gm26953/MIAT_exon5_1
AGGGGAGGAAGTAGGACAGAGGG	.*.......*.......*.*	4	0.125900376	0.27	chr5	1.4E+08	1.4E+08	+	intron:Gna12
AAGGGAGGAGGCAGGACAGTAGG	.*.......*.*.....*..	4	0.140621964	0.267206478	chr10	5.6E+07	5.6E+07	-	intergenic:Gm25602-Tbc1d32
ATGGGGGAATGAAGGAATGTAGG	.....*.*...*....*...	4	0.163959556	0.266666667	chr1	2.3E+07	2.3E+07	-	intron:Rims1
CTGGGAAGATTAAGGACTGTTGG	*.....*...**........	4	0.266829791	0.263736264	chr15	7E+07	7E+07	-	intergenic:Gm23987-Fam135b
GTGGGAGGTTGTGGGACAGTGGG	*.......*...*....*..	4	0.073380489	0.260869565	chr4	1E+08	1E+08	+	intron:Dab1
AAGAAAGGATTTAGGACTGTAGG	.*.**.....*.........	4	0.794051205	0.253846154	chr1	8.1E+07	8.1E+07	+	intron:Dock10
CTGGGAGTAGGCAGGACTGTGGG	*......*.*.*........	4	0.648306325	0.253759398	chr12	1.2E+08	1.2E+08	+	intron:Ptprn2
AGAGCAGGAGGTAGGACTGTGGG	.**.*....*..........	4	1.2395125	0.2475	chr4	6.8E+07	6.8E+07	+	intergenic:Gm11249-Gm11751
AGGTCAAGATGTAGGACTGTAGG	.*.**.*.............	4	0.891277473	0.24	chr5	1.5E+08	1.5E+08	+	intergenic:Cdk8-Wasf3
GGGGGAGGTTGTAGGAATGTAGG	**......*.......*...	4	0.372455417	0.2352	chr13	8.8E+07	8.8E+07	-	intergenic:Gm27044-Gm8526
TTGGGAGGCTCTAGAACTGTGGG	*.......*.*...*.....	4	0.136607805	0.230492197	chr16	1E+07	1E+07	+	intergenic:Emp2-Tekt5
ATGGGAGTCTGCAGGACTTTAGG	.......**..*......*.	4	0.135479128	0.22556391	chr16	8395956	8395978	+	intergenic:Gm26159-Gm24579
AGGGGAGGGAGTAGGATTGTGGG	.*......**......*...	4	0.343031439	0.224	chr11	5.4E+07	5.4E+07	-	intron:Rad50
AAGGGAAGATGTAGGAATGAGGG	.*....*.........*..*	4	0.183397224	0.222115385	chr18	2.1E+07	2.1E+07	+	intergenic:Dsg2-Gm16090/Ttr
ATGGGGAGATGTGGGAATGTGGG	.....**.....*...*...	4	0.088085272	0.217391304	chr12	6.3E+07	6.3E+07	-	intergenic:Spanxn4-Gm7763
ATGGGGAGATGTGGGAATGTGGG	.....**.....*...*...	4	0.088085272	0.217391304	chr12	6.3E+07	6.3E+07	-	intergenic:Spanxn4-Gm7763
CTGGGAGGAAGTGGGACTCTGGG	*........*..*.....*.	4	0.187783063	0.214790564	chr13	4E+07	4E+07	+	intergenic:Ofcc1-Gm9979

CTAGGAAGATGTAGGACGGTTGG	*.*...*..........*..	4	0.208990259	0.214285714	chr17	3.1E+07	3.1E+07	+	exon:Abcg1
AGGAGAGGTTGTAGGAATGTAGG	.*.*....*.......*...	4	0.372455417	0.21168	chr16	1.3E+07	1.3E+07	-	intergenic:Gm6327-Ercc4
ATTGGAAGATGTAGAACTCTAGG	..*...*.......*...*.	4	0.09001509	0.210953347	chr4	2.5E+07	2.5E+07	+	intergenic:Gm11894-Gm25975
AGAGGAGGATGTAGGATTATAGG	.**.............*.*.	4	0.189330488	0.21	chr16	2.7E+07	2.7E+07	-	intergenic:Gmnc-Ostn
ATGGGAGAATGTGGGAAAGTGGG	.......*....*...**..	4	0.041781429	0.202898551	chr14	4.8E+07	4.8E+07	+	intron:Gm6498
GTTGGAGGAAGTAGGATTGTGGG	*.*......*......*...	4	0.553566283	0.2	chr14	7.4E+07	7.4E+07	+	intergenic:Gm16409-Gm22164
ATGGGAGGATGTGAGACTAATGG	............**....**	4	0.010338516	0.196525621	chr3	2E+07	2E+07	-	intergenic:Gyg-Cpa3
AGGGGAGGATAGAGGATTGTGGG	.*........**....*...	4	0.166452825	0.196	chr1	1.7E+07	1.7E+07	-	intergenic:Gdap1-Gm25166
TGGGGAGGAGGTAGGAATGTGGG	**.......*......*...	4	0.56142625	0.196	chr15	2.6E+07	2.6E+07	+	intergenic:Gm5468-Myo10
CTTGGGGTATGTAGGACTGTAGG	*.*..*.*............	4	0.814229167	0.191326531	chr9	1.1E+08	1.1E+08	+	intron:Susd5
GTGGGAGGATGCTGGACAGTTGG	*..........**....*..	4	0.059088708	0.189473684	chr16	1.7E+07	1.7E+07	+	intergenic:Fgd4-Olfr19
ATGGAAGGACGCAGGACCGTTGG	....*....*.*.....*..	4	0.133501865	0.187669173	chr10	4.3E+07	4.3E+07	-	intergenic:Scml4-Sobp
GTGGGAGGGTGGAGGACTGAGGG	*.......*..*.......*	4	0.209660993	0.1875	chr11	8.2E+07	8.2E+07	+	exon:Ccl1
ATGGGAGGATTTAAAACAGTGGG	..........*..**..*..	4	0.005929041	0.180995475	chr14	5.3E+07	5.3E+07	-	intergenic:Trav6n-6-Trav12n-1
ATGGCAGGATGTGAGAATGTGGG	....*.......**..*...	4	0.033370636	0.179347826	chr14	2E+07	2E+07	+	intron:Gng2
ATGGGGGTAGGAAGGACTGTAGG	.....*.*.*.*........	4	0.374191978	0.178571429	chr1	4.1E+07	4.1E+07	-	intron:Slc9a2
ATGGGGGGGTGAAGGATTGTAGG	.....*..*..*....*...	4	0.100179289	0.177777778	chr6	2.4E+07	2.4E+07	-	intergenic:Cadps2-Gm24217
ATGGAAGGATGAATAACTGTGGG	....*......*.**.....	4	0.02810876	0.174013072	chr15	8.8E+07	8.8E+07	+	intron:Fam19a5
TTGGGAGGATGCAGAGCTGTGGG	*..........*..**....	4	0.035908784	0.171469397	chr8	2.7E+07	2.7E+07	+	intron:Poteg

TTGGGAGGTTCTAGGACTTTTGG	*.......*.*.......*.	4	0.178656992	0.171428572	chr6	1.5E+08	1.5E+08	+	intergenic:Klhl42-Gm7571
AAGAGAGGATGGAGGACTCTGGG	.*.*.......*......*.	4	0.245385	0.170689655	chr14	6.2E+07	6.2E+07	-	intergenic:Dleu2-Gm26969
ATGGGAGATTCTGGGACTGTGGG	.......**.*.*.......	4	0.171252683	0.167701864	chr9	1.1E+08	1.1E+08	+	intron:Col7a1
CTGGGAGGACTCAGGACTGTGGG	*........***........	4	0.359810011	0.166570272	chr1	1.6E+08	1.6E+08	+	intergenic:Prrx1-Gorab
CTGATGGGATGTAGGACTGTTGG	*..***..............	4	0.789491758	0.165306122	chr9	1.1E+08	1.1E+08	+	intron:Bsn
AGTGCAGGATGGAGGACTGTGGG	.**.*......*........	4	0.694060843	0.165	chr5	8367472	8367494	-	intron:Adam22
ATGGGGGCATGAAGGATTGTAGG	.....*.*...*....*...	4	0.163959556	0.164102564	chr4	8.8E+07	8.8E+07	+	intron:Mllt3
ATAGGAGGAGGTGGGACAGTGGG	..*......*..*....*..	4	0.109062622	0.163043478	chr14	6.1E+07	6.1E+07	+	exon:Spata13
ATGGGAGAAGGGGGGACTGTAGG	.......*.*.**.......	4	0.228837884	0.163043478	chr14	6.3E+07	6.3E+07	-	intron:Gata4
ATGGGTGGATCTAGAACTGAAGG	.....*....*...*....*	4	0.056406056	0.162064826	chr7	1.6E+07	1.6E+07	-	exon:Ccdc9
ATTGGAGGAAGAAGGATTGTAGG	..*......*.*....*...	4	0.258564504	0.16	chr2	4.8E+07	4.8E+07	+	intergenic:Gm25264-Gm25959
ATGGAAGGATCCAGGACTCTAGG	....*.....**......*.	4	0.129293046	0.157739176	chr13	1.1E+08	1.1E+08	-	intergenic:Htr1a-Dph3b-ps
ATAAGAGGAGGTAGGAATGTGGG	..**.....*......*...	4	0.52553761	0.1575	chr7	9.7E+07	9.7E+07	+	intron:Tenm4
AGGGGAGGAGGAAGGATTGTGGG	.*.......*.*....*...	4	0.276221715	0.1568	chr10	8.5E+07	8.5E+07	+	intergenic:Cry1-AC100386.1
AGGGGAGGAGGAAGGATTGTGGG	.*.......*.*....*...	4	0.276221715	0.1568	chr17	3.7E+07	3.7E+07	+	intergenic:H2-M5-Zfp57
ATAGGAGGAAGTAGGACGATGGG	..*......*.......**.	4	0.088771902	0.153061224	chr3	1.4E+08	1.4E+08	-	intergenic:Rap1gds1-Gm4862
GTGGGTGGATGAATGACTGTTGG	*....*.....*.*......	4	0.066667362	0.152380953	chr5	1.1E+08	1.1E+08	-	intron:Crybb2
CTGTGAGGAGGCAGGACTGTGGG	*..*.....*.*........	4	0.648306325	0.147641832	chr13	8.3E+07	8.3E+07	-	intergenic:Gm24295-Mef2c
TTTGGAGGATGTAGGACCTTTGG	*.*..............**.	4	0.101816651	0.142857143	chrX	1.6E+08	1.6E+08	+	intergenic:Gm26007-Gm7199

ATATGAGGATGAGGG ACTGTGGG	..**.......* *.......	4	0.26860154 6	0.14229249	chr1 9	5157 231	5157 253	-	intron:Pacs1
ATTGGAGGATGCAGG ACACTGGG	..*........*. ....**.	4	0.04742212 4	0.14156079 9	chr X	1E+0 8	1E+0 8	+	intron:Slc16a2
ATAGGAGGAGGAAG GATTGTAGG	..*......*.* ....*...	4	0.25856450 4	0.14	chr5	2.2E +07	2.2E +07	-	intergenic:Gm25459-n-R5s170
ATGGGAGTATGGGGG ACTTTTGG	.......*...* *.....*.	4	0.08581083 9	0.13586956 5	chr1 4	8.4E +07	8.4E +07	-	intergenic:Gm24774-Pcdh17
ATGGGAGTATGGGGG ACTTTGGG	.......*...* *.....*.	4	0.08581083 9	0.13586956 5	chr1 4	9351 961	9351 983	+	intergenic:Gm9800-Fhit
AAGGTAGGATGAAG GACAGTTGG	.*..*......* .....*..	4	0.152684	0.13538461 5	chr1 6	4.5E +07	4.5E +07	-	intergenic:Boc-Mir3081
ATTGGAGGATGGAGA ACTGAGGG	..*........*. .*....*	4	0.08583911 3	0.13235294 1	chr1 7	3.8E +07	3.8E +07	+	intergenic:Gm6726-Gm20410
ATTGGAGATTCTAGG ACTGTTGG	..*....**.*	4	0.45637881 3	0.12857142 9	chr4	7.2E +07	7.2E +07	+	intergenic:C630043F03Rik-Gm11235
ATTGGTGGATGGAGG ACTATAGG	..*..*.....* ......*.	4	0.14637951 4	0.12755102	chr8	3.1E +07	3.1E +07	+	intergenic:4933433F19Rik-Dusp26
CTGGGATGATGCAGC ACTGTTGG	*.....*....* ..*.....	4	0.13537146 5	0.12655014 2	chr1	1.6E +08	1.6E +08	+	intergenic:Dnm3-2810442N19Rik
GTGGGAGGCTGAAGC ACTGTGGG	*.......*..* ..*.....	4	0.12110097 3	0.12467532 5	chr6	7E+0 7	7E+0 7	+	exon:Igkv6-20
ATGGGAGGGGGAAG GATTGTGGG	........**.* ....*...	4	0.14549264 5	0.12444444 5	chr1 8	7.2E +07	7.2E +07	-	intron:Dcc
ACGGAAGGATGTAG AACTGGGGG	.*..*........ .*....*	4	0.18691584 5	0.12338111 7	chr5	5.1E +07	5.1E +07	-	intergenic:Gm22618-Gm22976
AGGGGAGGATGAAG CACAGTAGG	.*.........*. .*..*..	4	0.04091931 2	0.12218181 8	chr1 1	5.7E +07	5.7E +07	-	intron:Gria1
ATTGGAGGATGAGGG AATGTGGG	..*........* *...*...	4	0.10864762 6	0.12173913 1	chr1 3	8.9E +07	8.9E +07	-	intron:Edil3
ATGTGAGTGTGAAGG ACTGTTGG	...*...**.. *........	4	0.41031810 3	0.12121212 1	chr4	1.5E +08	1.5E +08	+	intergenic:Fbxo2-Ptchd2
ATGTGAAGAGGTAGG ACTTTTGG	...*..*..*.. ......*.	4	0.31373519 6	0.12121212 1	chr1 3	9360 723	9360 745	-	intergenic:Dip2c-Gm26601
CTGATAGGAGGTAGG ACTGTTGG	*..**....*.	4	1.31769578 3	0.11571428 6	chr X	9.2E +07	9.2E +07	-	intergenic:Mageb5-Gm14782
AAGGTCAGATGTAGG ACTGTAGG	.*..***....	4	0.53922287 1	0.11538461 5	chr9	1.2E +08	1.2E +08	-	intron:Ano10

ATGGGAGGGTGTGGGATTGAGGG	........*...*...*..*	4	0.054313087	0.114130435	chr15	1E+08	1E+08	+	intergenic:Krt80-Krt7
ATGGAAGGATTTAGGATTTTAGG	....*.....*.....*.*.	4	0.101174436	0.103703704	chr13	7.3E+07	7.3E+07	+	intergenic:Irx2-Irx4
ATGAGAGGATCTAGGAATGAAGG	...*......*.....*..*	4	0.141078919	0.10125	chr4	7E+07	7E+07	-	intergenic:Gm11404-Gm11221
CTGGGAGGCCTTAGGACTGTGGG	*.......***.........	4	0.446837229	0.100470958	chrX	9E+07	9E+07	+	intergenic:Gm6027-Gm4746
ATGGGAAGATCGAGGATTGTGGG	......*...**....*...	4	0.102729469	0.1	chr4	9.3E+07	9.3E+07	-	intergenic:Gm12642-Tusc1
ATAGGATGAGGTAGGAATGTGGG	..*...*..*......*...	4	0.358942188	0.1	chr8	9E+07	9E+07	-	intergenic:Gm24212-Tox3
AGGGGAGGATATAGGACAGGTGG	.*........*......*.*	4	0.075868305	0.098823529	chrX	1.3E+08	1.3E+08	+	intergenic:4921511C20Rik-Gm22636
AGGAGAGGATGTAGGGCAGTGGG	.*.*.........*.*..	4	0.053377333	0.096923077	chr11	3.2E+07	3.2E+07	+	intergenic:Nsg2-Gm12108
AGTGGAGGAAGTATGACTGTTGG	.**......*...*......	4	0.20338988	0.096	chr1	1.1E+08	1.1E+08	+	intergenic:2310035C23Rik-Tnfrsf11a
ATGGGAGGAGGTTGAACTTTCGG	.........*..*.*...*.	4	0.043049833	0.094117647	chr12	6.7E+07	6.7E+07	+	intergenic:MDGA2/Mdga2-Mdga2
ATGGCAGGATTTAGGATAGTAGG	....*.....*.....**..	4	0.062952983	0.094017094	chrX	1.5E+08	1.5E+08	-	intergenic:Gm8320-Gm15114
ATGGCAGGATTTAGGATAGTAGG	....*.....*.....**..	4	0.062952983	0.094017094	chrX	1.5E+08	1.5E+08	-	intergenic:Gm8303-Gm15107
ATGGCAGGATTTAGGATAGTAGG	....*.....*.....**..	4	0.062952983	0.094017094	chrX	1.5E+08	1.5E+08	-	intergenic:Gm15085-Gm15086
ATGGCAGGATTTAGGATAGTAGG	....*.....*.....**..	4	0.062952983	0.094017094	chrX	1.5E+08	1.5E+08	-	intergenic:Gm15085-Gm15086
ATGGCAGGATTTAGGATAGTAGG	....*.....*.....**..	4	0.062952983	0.094017094	chrX	1.5E+08	1.5E+08	-	intergenic:Gm8346-Gm15093
ATGGCAGGATTTAGGATAGTAGG	....*.....*.....**..	4	0.062952983	0.094017094	chrX	1.5E+08	1.5E+08	-	intergenic:Gm8366-Gm15100
ATGGCAGGATTTAGGATAGTAGG	....*.....*.....**..	4	0.062952983	0.094017094	chrX	1.5E+08	1.5E+08	-	intergenic:Gm8334-Gm15127
ATGGCAGGATGTAACACTGAGGG	....*........**....*	4	0.026365103	0.090401786	chr16	4.3E+07	4.3E+07	-	intron:Zbtb20
CGGGGAGGAAGTAGTACTGTGGG	**.......*....*.....	4	0.371023101	0.088163265	chr2	1.1E+08	1.1E+08	-	exon:Ccdc73

ATGGCAGGAAGTAG GGCAGTAGG	....*....*... ..*.*..	4	0.04667138 4	0.08634222 9	chr1 2	5.3E +07	5.3E +07	+	intron:Akap6
CTGGGAGGTTGCAGG ACTGGGGG	*.......*..* ....*	4	0.20966099 3	0.08597965 5	chr1 2	7.3E +07	7.3E +07	+	intergenic:D830013O20Rik-Tmem30b
ACGGGAGGATGGAG GATCGTTGG	.*.........*. ...**..	4	0.05878334	0.08571428 6	chr1 6	9.4E +07	9.4E +07	-	intergenic:Dopey2- 2310043M15Rik/Dopey2
CAGGGAGGATGTGG GACTGGGGG	**.......... *......*	4	0.26991206	0.08347151 6	chr1 6	3.2E +07	3.2E +07	-	intergenic:Nrros-Bex6
AAGGGAGGAAGTGG GACTGGAGG	.*.......*.. *......*	4	0.24858900 7	0.08347151 6	chr1	2.1E +07	2.1E +07	+	intergenic:Gm24723-6720483E21Rik
AAGGGATGCTGTTGG ACTGTGGG	.*....*.*... *.......	4	0.23106193 4	0.08288854	chr1 7	8210 086	8210 108	+	intergenic:Fgfr1op-Ccr6
AGGGGTGGATGTTGG ACTCTAGG	.*...*...... *.....*.	4	0.11677483 1	0.08068965 5	chr5	1.2E +08	1.2E +08	-	intergenic:Gm16338-Pitpnm2
ATGGGTGGAGGGAG GACTCTGGG	.....*...*.* ......*.	4	0.12980672 4	0.08004926 1	chr4	1.5E +08	1.5E +08	-	intron:Slc25a33
CTGGGAGGAAGTAG GGCTGAGGG	*........*... ..*...*	4	0.11048400 3	0.07947409 7	chr4	1.4E +08	1.4E +08	+	intron:Rap1gap
ATGGGAAGAAATAG GACTGCTGG	......*..**. ....*	4	0.21883455 4	0.07792207 8	chr1 0	9051 158	9051 180	-	intergenic:Gm26674/Sash1-Gm9930
ATGGGAAGATGGAG GAAGGTTGG	......*....*. ...**..	4	0.03627923 6	0.07777777 8	chr5	1.5E +08	1.5E +08	-	intron:Slc7a1
AGGGGAGGACTTAG GACTCTGGG	.*.......**. ......*.	4	0.25493855 6	0.07724137 9	chr9	5.5E +07	5.5E +07	-	intergenic:Cib2-Idh3a
TTGGGAGGATTTAGG ATCGTAGG	*.........*.. ...**..	4	0.06631047 5	0.07692307 7	chr4	1.1E +08	1.1E +08	-	intron:BC055111
AGGGGAGGAGGCAG GGCTGTGGG	.*.......*.* ...*....	4	0.11715469 7	0.07651821 8	chr1 7	2.6E +07	2.6E +07	-	intergenic:Sstr5-Sox8
AGGGGAGGATTGAG GATTGTAGG	.*........** ....*...	4	0.16645282 5	0.07538461 6	chr X	1.6E +08	1.6E +08	-	intron:Txlng
CTGGGAGGATCTAGG ACCCTAGG	*.........*.. ....**.	4	0.05731059	0.07057404 3	chr1 1	1.6E +07	1.6E +07	+	intergenic:Gm12010-Gm12011
ATGGAAGGATTTAGG ATTCTTGG	....*.....*.. ...*.*.	4	0.10117443 6	0.06973180 1	chr2	4.3E +07	4.3E +07	+	intergenic:Gm24350-Gm13464
ATGTGTGGATATATG ACTGTAGG	...*.*....*. .*......	4	0.07157974 6	0.06926406 9	chr1 0	2E+0 7	2E+0 7	+	intron:Pex7
AAGAGAGAATGTAG GACTGCGGG	.*.*...*.... ....*	4	0.69744718 3	0.06923076 9	chr1 1	6.9E +07	6.9E +07	+	intron:Alox8

TTGGGGGGAGGTAGGGCTGTGGG	*....*...*.....*....	4	0.151745495	0.068681319	chr1	1.3E+07	1.3E+07	-	intergenic:Ncoa2-AC121538.1
TTGGGAGGATGAAGAACTGCAGG	*.........*..*....*	4	0.091962596	0.068449198	chr5	1.4E+08	1.4E+08	-	intron:Baz1b
ATTGGAGGATGTGACACTGTGGG	..*.........***.....	4	0.022904218	0.066699605	chr8	1.1E+08	1.1E+08	-	intergenic:Wwox-Gm16116
GTGGGGGGATGTGGTACTGTCGG	*....*......*.*.....	4	0.09432084	0.066548359	chr17	4.4E+07	4.4E+07	-	intergenic:Gm24532-Rcan2
CTGGGAGGATGCTGCACTGTTGG	*..........**.*.....	4	0.07670389	0.066438824	chr6	1.2E+08	1.2E+08	-	intergenic:Rpl28-ps4-Gm9946
GTGGGGGGATGTAGGGTTGTGGG	*....*.........**...	4	0.063433242	0.064102564	chr2	6837162	6837184	-	intron:Celf2
ATGGGAGGAGGTGGGACCCTGGG	.........*..*....**.	4	0.031484206	0.062647248	chr7	1.1E+08	1.1E+08	-	intron:Cyb5r2
ATGGGAGGATGAAGAAATGGAGG	...........*..*.*..*	4	0.028894225	0.06200692	chr18	8.4E+07	8.4E+07	+	intergenic:Gm24720-Tshz1
ATGGGTGGATGAAGGGCTGAAGG	.....*.....*...*...*	4	0.03209161	0.061813187	chr15	7.1E+07	7.1E+07	+	intergenic:Gm23987-Fam135b
ATGTGAGGACGTAGGATAGTAGG	...*.....*......**..	4	0.104467922	0.0603367	chr16	4.7E+07	4.7E+07	-	intergenic:Pvrl3-Gm6912
AGGGGAGGATCGAGGACGGTTGG	.*........**.....*..	4	0.08473962	0.06	chr6	4747105	4747127	-	exon:Sgce
ATGGGAGGACCCAGCACTGTGGG	.........***..*.....	4	0.087951801	0.059056733	chr4	1.4E+08	1.4E+08	+	intergenic:Myom3-Gm13000
ATGTGATGTTGTAGGATTGTGGG	...*..*.*.......*...	4	0.241506693	0.058181818	chr5	2E+07	2E+07	+	intron:Magi2
AAGGGAGGAGGGAGCACTGTCGG	.*.......*.*..*.....	4	0.182543366	0.057692308	chr13	1E+08	1E+08	-	intron:Mccc2
ATGGAAGGATGAAGGAATGGAGG	....*......*....*..*	4	0.125064555	0.057098039	chr7	1.4E+08	1.4E+08	+	intergenic:Gm9358-Jakmip3
ATGGGAGGATGAAGGATGCTGGG	...........*....***.	4	0.015962717	0.055785441	chr4	1.3E+08	1.3E+08	-	intron:Grik3
ATGGGTGGATGTACCACAGTAGG	.....*.......**..*..	4	0.007117689	0.055658627	chr14	1.2E+08	1.2E+08	-	intergenic:Clybl-Gm5089
AGGGGAGGATGAAGGAATGGAGG	.*.........*....*..*	4	0.132110445	0.055341176	chr5	1.3E+08	1.3E+08	-	intron:Glt1d1
CTGATAGGAAGTAGGACTGTAAG	*..**....*..........	4	0.263539157	0.051428571	chr3	1.8E+07	1.8E+07	-	intergenic:Cyp7b1-Gm6369

ATGGGAGGCTTTTAG ACTGTTGG	........*.*. **......	4	0.02551665	0.04945054 9	chr4	3.9E +07	3.9E +07	-	intergenic:Gm12381-Gm25581
AAGGGAGGAGGAAG TACTGTGGG	.*.......*.* ..*.....	4	0.18254336 6	0.04835164 8	chr6	2.8E +07	2.8E +07	+	intron:Zfp800
ATGGTTGGATGTTAG ACTGTGGG	....**...... **......	4	0.04991236 3	0.04821428 6	chr1	1.2E +08	1.2E +08	+	intergenic:Gm25578-Actr3
ATGGCAGGTTGTTGG ACGGTGGG	....*...*... *....*..	4	0.06966502 1	0.04714285 7	chr1 8	7.9E +07	7.9E +07	-	intergenic:Setbp1-Gm25824
ATGGGAGGAGGAAG GACAGGAGG	.........*.*. ....*.*	4	0.05298737 3	0.04705882 3	chr1 1	8.5E +07	8.5E +07	-	intron:Bcas3
GTGGGAGGATGTTGG AAGGTTGG	*...........* ...**..	4	0.04623811 5	0.04666666 7	chr1 5	8.5E +07	8.5E +07	-	exon:Phf21b
AAGTTAGGAGGTAGG ACTGTAGG	.*.**....*.	4	1.25711206 9	0.04615384 6	chr1 5	4.4E +07	4.4E +07	-	intergenic:Tmem74-Trhr
AAGAGAGGATGTTGG GCTGTGGG	.*.*........ *..*....	4	0.10005664 6	0.04393491 1	chr1 1	3E+0 7	3E+0 7	+	intron:Eml6
ATGGGAGGATGGAG GAAGGAAGG	...........*.. ..**.*	4	0.02113159 7	0.04375	chr1	3.3E +07	3.3E +07	-	intergenic:Gm24901-Gm25792
ATCAGAGGATGTGGG GCTGTAGG	..**........ *..*....	4	0.09865585 3	0.04341394 4	chr6	1.2E +08	1.2E +08	+	exon:Kdm5a
ATGGGGGGGGGTAG GACTGGAGG	.....*..**.. ....*	4	0.21340215 3	0.04201680 7	chr1 3	1E+0 8	1E+0 8	+	intron:Mast4
ATACGAGGATTTAGC ACTGTAGG	..**......*. ..*.....	4	0.22045056 6	0.04164952 7	chr1 2	1.1E +08	1.1E +08	-	intron:Atg2b
GTGGGAGGATGTATC ACTGAAGG	*........... **....*	4	0.02785046 1	0.04090909 1	chr9	5.4E +07	5.4E +07	+	intergenic:Cyp19a1-1700104A03Rik
ATGACAGGATGTTGG GCTGTAGG	...**....... *..*....	4	0.10005664 6	0.04079670 3	chr1 5	7.4E +07	7.4E +07	-	intergenic:Gm6569/Mroh5-Gm7935
CTGTTAGGATCTAGG ACTGTGGG	*..**.....*	4	0.79405120 5	0.04007421 2	chr5	1.1E +08	1.1E +08	+	intron:Ssh1
ATTGCAGGGTGTAGT ACTGTGGG	..*.*...*... ..*.....	4	0.24269422 7	0.03741496 6	chr Y	3969 512	3969 534	+	intergenic:Gm8521-Gm20918
AATGGAGGATGGAG GACTGGGGG	.**........* ....*	4	0.33833999 8	0.03733031 7	chr1 0	3.8E +07	3.8E +07	+	intergenic:Gm26535-Gm24710
ATGGGAGGGTGGAG GGCTGAGGG	........*..*. ..*...*	4	0.03084795 2	0.03605769 2	chr9	2.2E +07	2.2E +07	-	intron:Cnn1
ATGGGAAGATCTAGG ATTGGAGG	......*...*.. ...*..*	4	0.09147807 1	0.03529411 8	chr1 7	1.3E +07	1.3E +07	+	intron:Sod2

ATGGGAGGATATTCCACTGTGGG	..........*.***.....	4	0.011192246	0.035064935	chr6	8.5E+07	8.5E+07	+	intergenic:Spr-ps1-Emx1
ATGGGAGGAGGAAGGATTGGGGG	.........*.*....*..*	4	0.104082339	0.032941176	chr8	8.8E+07	8.8E+07	-	intergenic:Brd7-Nkd1
AGCTGAGGATGTAGCACTGTTGG	.***..........*.....	4	0.397208228	0.032040687	chr7	5.2E+07	5.2E+07	+	intergenic:Gm22211-Gm6181
AGCTGAGGATGTAGCACTGTGGG	.***..........*.....	4	0.397208228	0.032040687	chr14	1.2E+08	1.2E+08	-	intergenic:Tm9sf2-Clybl
ATGGGAGAATGCAGGAGTGATGG	.......*...*....*..*	4	0.118732172	0.031346749	chr1	1.9E+08	1.9E+08	+	intron:Eprs
ATGGGAGCATGTTGGAAGGTAGG	.......*....*...**..	4	0.041781429	0.028717949	chr12	1.1E+08	1.1E+08	+	intergenic:BC022687-Cdca4
AGCGGAGGATGGAGGACTGGAGG	.**........*.......*	4	0.338339998	0.028506787	chrX	1.2E+07	1.2E+07	+	intergenic:Gm14513-Gm26314
ATTGGAGTATGCAGGACTGCCGG	..*....*...*.......*	4	0.320295198	0.026913876	chr7	6.6E+07	6.6E+07	+	intron:Aldh1a3
GGGTTAGGATGTAGGACTGTTAG	**.**...............	4	0.260989011	0.023757576	chr7	1.4E+08	1.4E+08	-	intergenic:Tcerg1l-Mapk1ip1
GTGGGTGGATGTAGGAGTGATGG	*....*..........*..*	4	0.162452885	0.023634454	chr16	1.4E+07	1.4E+07	+	intron:Myh11
ATTGTAGGATGTAGGATGGTAGG	..*.*...........**..	4	0.117805637	0.023333333	chrX	4.2E+07	4.2E+07	-	intergenic:Gria3-Gria3/Gm22413
ATGGCAGAATGGAGGAGTGTGGG	....*..*...*....*...	4	0.28472943	0.023109244	chr15	5.9E+07	5.9E+07	-	intron:Fer1l6
ATGGAAGGGTGTAGGAGTTTGGG	....*...*.......*.*.	4	0.111383028	0.022657952	chr4	9.4E+07	9.4E+07	-	intron:Gm12649
GTGGGAGGCTGTGGGAGTGTGGG	*.......*...*...*...	4	0.144140246	0.021921812	chr4	1.2E+08	1.2E+08	-	intron:Gm12923
ATAGAAGGATGTAGGAGTGAGGG	..*.*...........*..*	4	0.250637503	0.021507353	chr4	3.1E+07	3.1E+07	+	intergenic:4930556G01Rik-Gm25705
TTGGGAGGAGGTAGGAATGCAGG	*........*......*..*	4	0.247304309	0.021212121	chr2	1.5E+08	1.5E+08	+	intergenic:Gm14216-Gm14214
GTGGGTGGATGGAGGAGTGTGGG	*....*.....*....*...	4	0.181448575	0.021008403	chrX	1.1E+08	1.1E+08	-	intergenic:Gm6377/Sh3bgrl-Gm14868
ATGGGAGGGTGTCAGGCTGTGGG	........*...**.*....	4	0.008271443	0.020242915	chr12	5.7E+07	5.7E+07	-	intergenic:Nkx2-9-Gm15524
AATGGAGGATGTATGACTGGTGG	.**..........*.....*	4	0.102464755	0.019909502	chr7	9.9E+07	9.9E+07	+	intergenic:Prkrir-Wnt11

TTTGGAGGATGTGGGAGTGTTGG	*.*.........*...*...	4	0.232606028	0.019181586	chrX	2.3E+07	2.3E+07	-	intergenic:Gm26131-Klhl13
ATTGGGTGATGTAGGACTGCTGG	..*..**............*	4	0.269005651	0.018552876	chr2	3.2E+07	3.2E+07	+	intergenic:Prrc2b-AL808027.1
ATTGGAGGAGGTATCACTGTGGG	..*......*...**.....	4	0.054508488	0.018181818	chr13	5.9E+07	5.9E+07	+	intergenic:Slc28a3-Ntrk2
ATGGGATGATGTGGGAATGCTGG	......*.....*...*..*	4	0.063787412	0.015810277	chr3	1E+08	1E+08	+	intron:Casq2
ATGGTAGAATGTAGCGCTGTGGG	....*..*......**....	4	0.065950602	0.015734266	chr1	8.3E+07	8.3E+07	+	intergenic:Slc19a3-Ccl20
ATGGGTGGAGGTAGGATTGCTGG	.....*...*......*..*	4	0.134467805	0.015151515	chr16	4327553	4327575	-	intron:Adcy9
ATGGGAGAATGTACGAGTGAGGG	.......*.....*..*..*	4	0.035957508	0.014180672	chr5	3218881	3218903	-	intergenic:Gm8715-Gm15772
ATGGGAAGATGATGGAGTGTTGG	......*....**...*...	4	0.071632981	0.014117647	chr4	1.2E+08	1.2E+08	-	intergenic:Mast2-Gm12950
ATGGGAGGATGGAGGATGGGAGG	...........*....**.*	4	0.021131597	0.01372549	chr3	1.2E+08	1.2E+08	+	intergenic:Mir760-Bcar3
ATGGGAGGATGGAGGATGGGAGG	...........*....**.*	4	0.021131597	0.01372549	chr18	8.4E+07	8.4E+07	+	intergenic:Gm24720-Tshz1
ATGGGAGGATGGAGGATGGGAGG	...........*....**.*	4	0.021131597	0.01372549	chr3	1.2E+08	1.2E+08	+	intergenic:Mir760-Bcar3
ATGGGAGGATGGAGGATGGGAGG	...........*....**.*	4	0.021131597	0.01372549	chr3	1.2E+08	1.2E+08	+	intergenic:Mir760-Bcar3
ATGGGAGGATGGAGGATGGGAGG	...........*....**.*	4	0.021131597	0.01372549	chr3	1.2E+08	1.2E+08	+	intergenic:Mir760-Bcar3
ATGGGAGGAGGTTGGGATGTGGG	.........*..*..**...	4	0.032216187	0.013461538	chr17	4.6E+07	4.6E+07	+	intron:Tmem63b
ATGTGAGGATGTAGAAGAGTTGG	...*.........*.**..	4	0.030398918	0.013421411	chr17	4315096	4315118	-	intergenic:Nox3-Gm23186
ATGGGAGGGTGGGGGAGTGTGGG	........*..**...*...	4	0.061135346	0.012787724	chr9	1E+08	1E+08	-	intron:Stag1
ATGGGAGGGTGGGGGAGTGTGGG	........*..**...*...	4	0.061135346	0.012787724	chr3	1.6E+08	1.6E+08	+	intergenic:Gm23038-4930570G19Rik
ATGGGAGGATGGCTGACTCTGGG	...........***....*.	4	0.012197961	0.012583182	chr10	8E+07	8E+07	+	exon:Cirbp
ATGGGTGGAGGGAGGAGTGTAGG	.....*...*.*....*...	4	0.151006751	0.010504202	chr2	1.2E+07	1.2E+07	-	intergenic:Pfkfb3-Rbm17

ATGGGAGGAGGGAGGAGAGTAGG	.........*.*....**..	4	0.046671945	0.009803922	chrX	6.9E+07	6.9E+07	-	intergenic:Gm14700-Gm14705
ATGGTAGGATGGAGGACAGCTGG	....*......*.....*.*	4	0.063669228	0.009090909	chr9	3.3E+07	3.3E+07	+	intergenic:RP24-86O15.2-Gm10698
TTGGCAGGATGTAGGGGTGTGGG	*...*.........**...	4	0.104848333	0.008888171	chr4	1.8E+07	1.8E+07	-	intron:Mmp16
ATGGGAGGAAGCAGGAGTGGGGG	.........*.*....*..*	4	0.104082339	0.008429378	chr12	1.1E+08	1.1E+08	+	intron:D430019H16Rik
CTGGGAGGATGGAGGAGGGTAGG	*..........*....**..	4	0.05878334	0.008403361	chrX	1.1E+07	1.1E+07	+	intergenic:Mid1ip1/Gm14493-Gm14473
ATGAGAGGATGTCAGAGTGTTGG	...*........**..*...	4	0.033370636	0.008359133	chr15	9.5E+07	9.5E+07	-	intron:Tmem117
ATGGTAGGATGTGGGAGTTTGGG	....*.......*...*.*.	4	0.070548661	0.007672634	chr14	1.2E+08	1.2E+08	+	intergenic:Hs6st3-Oxgr1
GTGGGAGGATGTGGGGGTGTGGG	*...........*..**...	4	0.040576305	0.007377533	chr2	5045493	5045515	+	intron:Optn
TTGGGAGGATGTAGGAGAGGGGG	*..............**.*	4	0.052629364	0.006920415	chr2	7E+07	7E+07	+	intron:Ppig
GGGTTAGGATGTAGGACTGTTGA	**.**...............	4	0.260989011	0.006363636	chr13	8.9E+07	8.9E+07	+	intron:Edil3
AGGGGAGGATGTAGGAGAGGAGG	.*.............**.*	4	0.052629364	0.005813149	chr11	3.2E+07	3.2E+07	-	intergenic:Bod1-D630024D03Rik
GTGGGAGGACGTAGGAGTGGGGG	*........*......*..*	4	0.247304309	0.005536332	chr13	5.8E+07	5.8E+07	-	intron:Klhl3
ATGGGAGGATGAGGGAGTGGTGG	...........**...*..*	4	0.041724123	0.005415977	chr17	6.1E+07	6.1E+07	-	intergenic:Gm5496-Gm4518
ATGGGTTGATGTAGGGGTGTGGG	.....**........**...	4	0.03914901	0.004617231	chr13	1.6E+07	1.6E+07	-	exon:Gli3
ATGGGAGGAGGTGGGAGTGGAGG	.........*..*...*..*	4	0.081869645	0.003384986	chr8	1.2E+08	1.2E+08	-	intron:2310022B05Rik
ATTGGAGGATGTAGGTTTCTGGG	..*............**.*.	4	0.032564844	0	chr12	1.1E+08	1.1E+08	-	intergenic:Cep170b-Pld4
ATTGGAAGATGTAGCCCTGTTGG	..*...*.......**....	4	0.046662434	0	chr2	5.3E+07	5.3E+07	-	intron:Fmnl2
ACGGGAGGCTGCAGGCCTGTGGG	.*......*..*...*....	4	0.07772152	0	chr17	2.5E+07	2.5E+07	-	exon:Mapk8ip3
ATGAGAGGATGCATGTCTGTTGG	...*.......*.*.*....	4	0.018953366	0	chr11	1.2E+08	1.2E+08	-	intron:Aatk

ATGGCAGAATGGAG GTCTGTCGG	....*..*...* ...*....	4	0.12107349 4	0	chr1 3	1.1E +08	1.1E +08	+	intron:4930544M13Rik
ATGGCAGGATGTAGA CCGGTCGG	....*......... **.*..	4	0.01358081 5	0	chr9	4.4E +07	4.4E +07	+	intergenic:Gm23326-Thy1
ATGGGACTATGTAGG TCTGCAGG	......**..... ..*...*	4	0.07363626 2	0	chr1 7	8.2E +07	8.2E +07	+	intergenic:Rpl31-ps25-AC135509.1
TTGGGATGATGGAGG CCTGTTGG	*.....*....* ...*....	4	0.09151380 4	0	chr1 2	8.6E +07	8.6E +07	-	exon:Mfsd7c
ATAAAAGGATGTAGG TCTGTGGG	..***....... ...*....	4	0.25492468 4	0	chr5	4086 116	4086 138	+	intron:Cyp51
ATGGGAGGAAGTAA GCTTGTGGG	.........*... *.**...	4	0.01240364 8	0	chr1	3.6E +07	3.6E +07	+	intergenic:Gm25096-Gm25634
ATGCTAGGGTGTAGG TCTGTGGG	...**...*... ...*....	4	0.15797057	0	chr6	9E+0 7	9E+0 7	-	intron:Ccdc37
AAGGGAGGATGAAG ATCTGTGGG	.*.........*. .**....	4	0.03409061 8	0	chr1	1.5E +08	1.5E +08	+	intergenic:Gm9931-Gm22966
ATGGGATGAAGTAGG TCTGAAGG	......*..*... ..*...*	4	0.06781899 7	0	chr2	1.5E +08	1.5E +08	-	intergenic:A530006G24Rik-Gm25516
GTGGGAGGATGTATG TGTGTTGG	*........... *.**...	4	0.01562240 2	0	chr3	8.7E +07	8.7E +07	-	intron:Kirrel
ATTAGAGGATGTAGC CCTGTTGG	..**......... .**....	4	0.06831981 5	0	chr1	2.3E +07	2.3E +07	+	intron:Rims1
ATGGGATGATGTAGG TCTAGAGG	......*....... .*..**	4	0.02319542 2	0	chr1 5	6.7E +07	6.7E +07	-	intergenic:Lrrc6-Tmem71
ATGGGAGGAAGTAG GTGTGGGGG	.........*.... .**..*	4	0.03638650 9	0	chr5	1.2E +08	1.2E +08	-	intron:Vsig10
AGCGGAGGATGTAG GTCAGTAGG	.**.......... ..*.*..	4	0.05263005 1	0	chr2	1.5E +08	1.5E +08	+	intergenic:A530006G24Rik-Gm25516
ATGGGAGGTTTTAGG TCTTTGGG	........*.*.. ..*..*.	4	0.02628625 5	0	chr1 6	3.2E +07	3.2E +07	+	intergenic:Bdh1-Gm26053
GTGGGTGTATGTAGG CCTGTGGG	*....*.*.... ...*....	4	0.16476166 7	0	chr3	8.5E +07	8.5E +07	+	intron:Fbxw7
ATGGAAGGATGGAG GCCAGTGGG	....*......*. ..*.*..	4	0.02493194 4	0	chr7	3.5E +07	3.5E +07	-	intron:Pepd
ATGGGAGGATGTTGG TGTCTGGG	............*. .**.*.	4	0.01101856 6	0	chr1 8	7.4E +07	7.4E +07	+	intergenic:Ccdc11-Myo5b
ATGGGTGGGTGTTGG CCTGTGGG	.....*..*... *..*....	4	0.03520396	0	chr X	1.7E +08	1.7E +08	-	intron:Glra2

ATGGAAGGAGGTAGTCCTGTGGG	....*....*....**....	4	0.060740505	0	chr11	1.2E+08	1.2E+08	-	intron:Rbfox3
CTGGGAGGAAGTAGGCCTATGGG	*........*....*..*.	4	0.083459139	0	chr8	7.3E+07	7.3E+07	+	intergenic:Nwd1-Sin3b
ATTGGAGGATGTAGGTCCCTTGG	..*...........*.**.	4	0.016578466	0	chr13	4.1E+07	4.1E+07	-	intergenic:Elovl2-Smim13
ATGGGAGGATGTATGCCTACTGG	.............*.*..**	4	0.004594896	0	chr8	1E+08	1E+08	+	intergenic:Gm8730-Cdh5
GTGGGAGAATGTATGCCTGTTGG	*......*.....*.*....	4	0.040577667	0	chr12	8E+07	8E+07	+	intergenic:Rad51b-Zfp36l1/Gm26669
AGGTGAGGATGTAGGCCAGTTGG	.*.*.........*.*..	4	0.053377333	0	chr2	2E+07	2E+07	-	intergenic:Etl4-Gm13335
CTGAGAGGATGTTGGCCTGTCGG	*..*........*..*....	4	0.105393	0	chr7	1.2E+08	1.2E+08	+	exon:Ern2
GTGGGAGGATGGAGGTCTGCTGG	*..........*...*...*	4	0.05902077	0	chr5	4.1E+07	4.1E+07	-	intergenic:Gm23022-Rab28
ATGGGAGGATGTTGGTCCATGGG	............*..*.**.	4	0.005609452	0	chr12	8E+07	8E+07	+	intergenic:Rad51b-Zfp36l1/Gm26669
ATGGGAGAAAGTAGGTCTATTGG	.......*.*.....*..*.	4	0.071392758	0	chr13	4.6E+07	4.6E+07	-	intergenic:Stmnd1-Rbm24
TTGGGAGCAGGTAGGCCTGTGGG	*......*.*.....*....	4	0.250819	0	chr8	7.7E+07	7.7E+07	+	intergenic:Gm7069-Gm10649
ATGGGAGGATGTCGGTGTCTCGG	............*..**.*.	4	0.011018566	0	chr15	7.7E+07	7.7E+07	-	intergenic:Mb-Apol6
TTGGGAGGATGTAGAACTTTTGG	*.............*...*.	3	0.302067797	0.627450981	chr19	2.4E+07	2.4E+07	+	intergenic:Pip5k1b-4930418C01Rik
CTGGGAGGATGTAGAACTTTTGG	*.............*...*.	3	0.302067797	0.537815126	chr6	4.3E+07	4.3E+07	+	intergenic:Olfr455-Fam115e
ATGGGAGGATATAGAAATGTTGG	..........*....*.*...	3	0.145653695	0.439215687	chrX	1E+08	1E+08	+	intergenic:Gm14828-Mir676
ATGGGAGGACAAAGGACTGTAGG	.........***........	3	0.583428198	0.426666666	chr19	2.2E+07	2.2E+07	-	intergenic:Gm22506-RP23-448C3.1
ATGAGAGCATGTGGGACTGTTGG	...*...*....*.......	3	1.034177215	0.361204013	chrX	5.1E+07	5.1E+07	+	intergenic:Gm14621-Hs6st2
ATGGGAGGGTGTGGGACTTTTGG	........*...*.....*.	3	0.20965854	0.289855073	chr4	3.7E+07	3.7E+07	+	intergenic:Gm23314-Lingo2
CTGGGAGGATGGAGGACTGATGG	*..........*.......*	3	0.734108475	0.241071429	chr7	1.1E+08	1.1E+08	-	intron:Tead1

ATGGGAGGATGTAGG AATAAAGG	....*.**	3	0.117321346	0.1875	chr4	1.3E+08	1.3E+08	+	intron:Wdtc1
ATGGGAGGGTGGAG GATTGTGGG	........*..*. ...*...	3	0.309279435	0.155555556	chr3	7.8E+07	7.8E+07	-	intergenic:Gm23644-Gm15442
AGAGGAGGAAGTAG GACTGTAAG	.**......*..	3	0.485344979	0.14	chrX	1.6E+08	1.6E+08	+	intron:Cnksr2
ATGTGAGGATTCAGG ACTGTTGG	...*......**	3	0.729696203	0.13249908	chr19	2.3E+07	2.3E+07	+	intron:Trpm3
ATGTGAGGATTTAGA ACTGTTGG	...*......*.. .*.....	3	0.418675556	0.131633073	chr7	1.1E+08	1.1E+08	-	intergenic:Gm9105-Stk33/1700095J03Rik
ATGGAGGGATGTAGG GCTGTGGG	....**....... ..*....	3	0.29290963	0.119047619	chr19	3E+07	3E+07	+	intergenic:Ranbp6-Il33
ACAGGAGGATGTGG GACTGTCAG	.**......... *.......	3	0.214816533	0.108695652	chr1	1.2E+08	1.2E+08	+	intergenic:Dpp10-Gm10543/Dpp10
AAGGAAGGAGGTAG GACTGTGAG	.*..*....*..	3	0.492236287	0.095061728	chr3	8.8E+07	8.8E+07	-	intron:Slc25a44
ATGGGAGAATGTAGG GTTGTGGG	.......*...... .**...	3	0.176959212	0.08974359	chr11	1E+07	1E+07	+	intergenic:Eif3s6-ps1-Gm11995
ATGGGAGTCAGTAGG ACTGTCAG	.......***..	3	0.26109619	0.079365079	chr7	5.2E+07	5.2E+07	-	intergenic:Gm22211-Gm6181
AGGGGAAGATGTAG GACTGCTGG	.*....*...... ......*	3	1.01909774	0.076363636	chr4	1.4E+08	1.4E+08	-	exon:Emc1
ATGTGTGGATGTATG ACTGTGGG	...*.*....... *......	3	0.253741481	0.069264069	chr4	1.2E+08	1.2E+08	-	intron:Ndufs5
ATGGTAGGATGTTGG ACTTTGGG	....*.......* .....*.	3	0.38411194	0.06	chr2	7.8E+07	7.8E+07	+	intron:Gm14461
ATGGGAGGATGTAGG AAAGGAGG	....**.*	3	0.072999949	0.054901961	chr10	9.6E+07	9.6E+07	+	intergenic:Gm24433-Eea1
AAGGAAGGATGTAG GAGTGTAGG	.*..*........ ...*...	3	1.213101161	0.043137255	chr3	5.4E+07	5.4E+07	+	intron:Frem2
CTGGAAGGATGAAG GACTGTAGA	*...*......*	3	0.276977778	0.041269841	chr13	1.1E+08	1.1E+08	+	intron:Elovl7
ATGGGAGGAGGTAG GACTACAGG	.........*.... ....**	3	0.34053054	0.032467532	chr16	9.4E+07	9.4E+07	-	intron:Ttc3
ATGGGAGCATGTAGG CCCGTAGG	.......*...... .*.*..	3	0.094893037	0	chr4	8.2E+07	8.2E+07	-	intergenic:Gm5860-Nfib
ATGGAAGGATGTAGG CCAGTGGG	....*......... .*.*..	3	0.100239124	0	chr10	8.1E+07	8.1E+07	-	exon:Adat3/Scamp4

CTGGGGGGATGTAGG TCTGTGGG	*....*....... ..*....	3	0.32788391 4	0	chr8	9.4E +07	9.4E +07	-	exon:Nlrc5
GTGGGAGGAAGTAG GTCTGTGGG	*........*... ..*....	3	0.49914228 9	0	chr1 7	8.2E +07	8.2E +07	-	intergenic:Rpl31-ps25-AC135509.1
ATGGGTGGATGTAGG ACTATTGG	.....*........ ....*.	2	2.10518895 3	0.51020408 1	chr1	9E+0 7	9E+0 7	+	intron:Iqca

Table S4. Summary of significant fold-changes in reference to the tested parameters in CatWalk gait analysis affected by *Adnp* knockdown, NAP treatment and sex.

Tested parameters		Genotype Effect G68 vs. Poly T		NAP Treatment Effect G68 NAP vs. G68		Sex Effect Females vs. Males		
		**Males**	**Females**	**Males**	**Females**	**Poly T**	**G68**	**G68 NAP**
**Run Characterization**	Run duration				0.77			
	Cadence	1.20	0.81			1.27		
**Interlimb Coordination**	Swing speed-RF		0.77		1.174			
	Swing speed-LF		0.74		1.23			
	Swing speed-LH		0.78		1.40		0.75	
	Body speed-RF		0.75		1.20			
	Body speed-LF		0.74		1.21			
	Body speed-RH		0.75					
	Body speed-LH		0.72		1.24			
	Step cycle-RF		1.30			0.826		
	Step cycle-LF					0.80		
	Step cycle-RH		1.35			0.792		
	Step cycle-LH	0.81	1.32		0.82	1.12	0.88	

	BOS- front paws						0.87	
	BOS- hind paws		0.84		1.10			
	Step Sequence				0.913			
	Support Three	0.85						
	Support Diagonal	1.29						
	Support Lateral							9.12
**Temporal Parameters**	Single Stance-RF		1.33			0.84		
	Single Stance-RH		1.27		0.82		1.20	
	Initial Dual Stance-RF	0.58	1.50			0.55		
	Initial Dual Stance-RH		1.87			0.47		
	Terminal Dual Stance-RH					0.52		
	Terminal Dual Stance-LH	0.60				0.61		
	Max Intensity AT		0.90			1.10		
**Spatial Parameters**	Mean Intensity Of The 15 Most Intense Pixel-RF		0.93			0.94	0.91	0.92
	Mean Intensity Of The 15 Most Intense Pixel-LF		0.93		1.10		0.91	
	Mean Intensity Of The 15 Most Intense Pixel-RH							0.82
	Mean Intensity Of The 15 Most Intense Pixel-LH					0.86		0.86
	Print Width-RH					0.86		
	Print Width-LH		0.92				0.91	

Supplemental Figures:

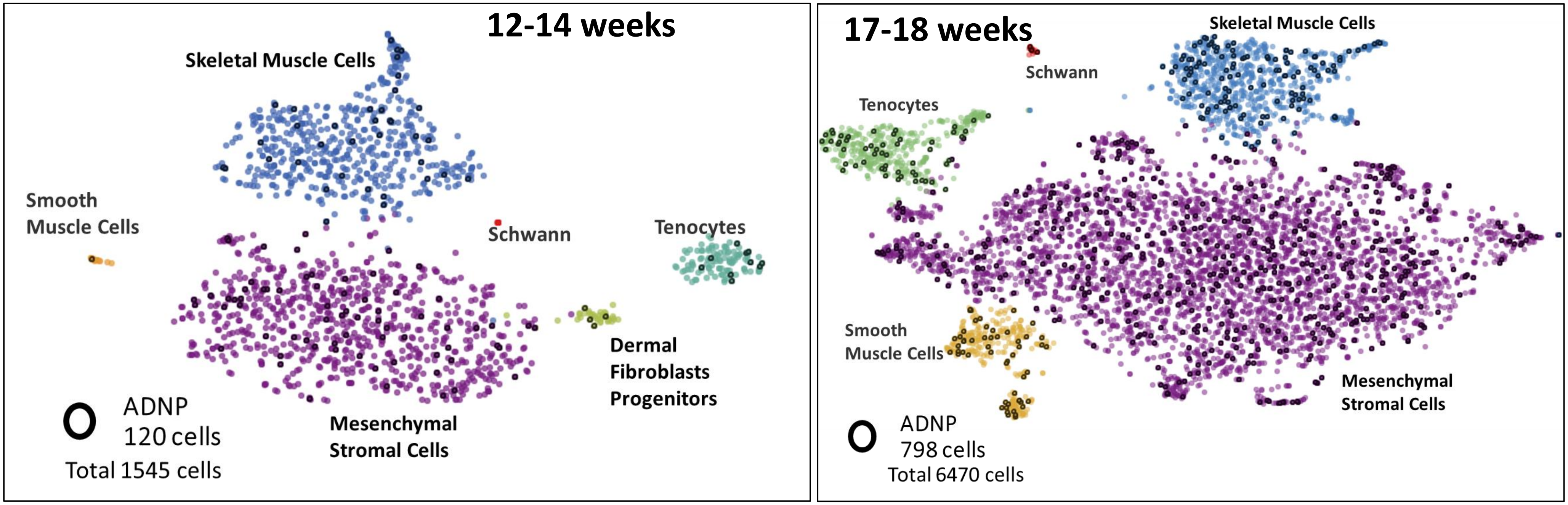

Figure S1. Single human muscle cell ADNP cellular distribution at different time points during gestation.

As in Figure 1, assessing human embryo limbs at 12-14 and 17-18 weeks of gestations.

Expression levels

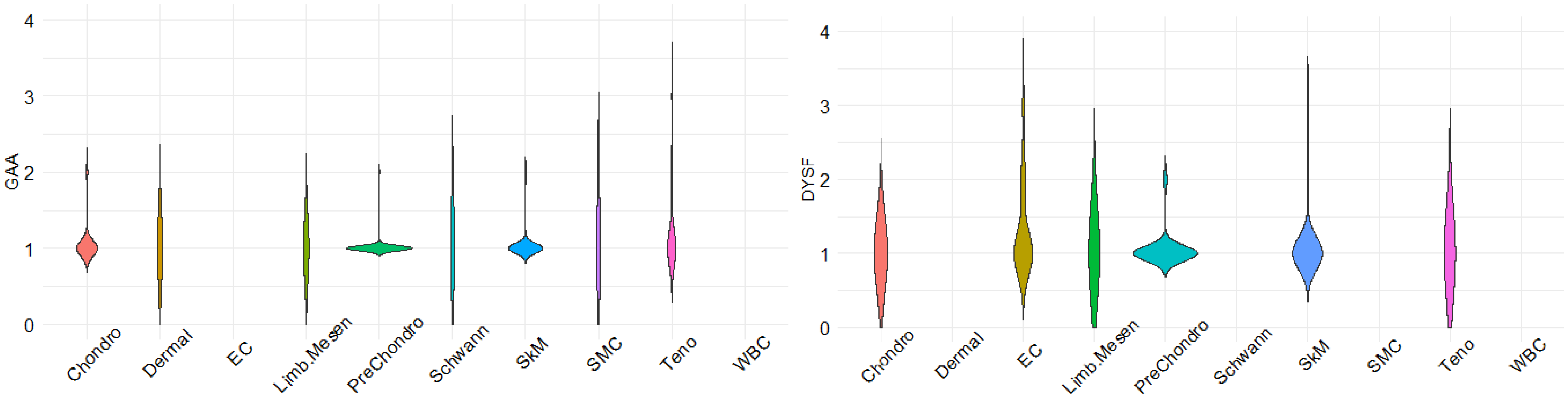

Figure S2 Single cell specific gene transcriptomes of hind limbs from human fetuses of 9 weeks

Violin graphs as in Figure 1, representative transcripts as per diseases depicted in Figure 2.

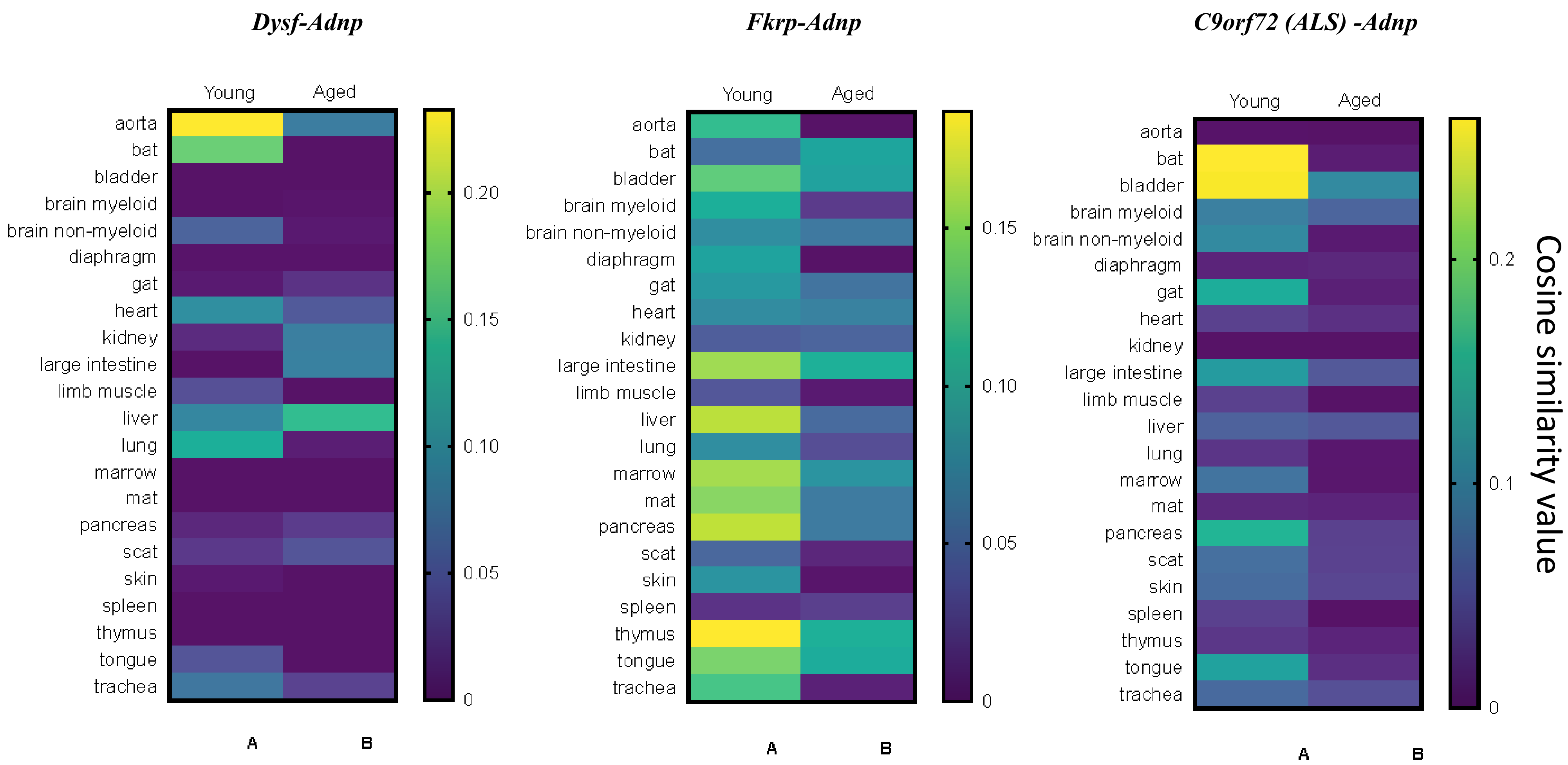

Figure S3. Overall systemic loss of correlation of expression of *Adnp* and disease genes at the single cell level. Heatmaps plotting cosine similarity values (indicating co-expression at the single cell level) for the genes of interest (indicated above each heatmap) and *Adnp* in young (3 months) and aged mice(18 and 24 months) in 22 tissues (indicated in rows). GAT, SCAT, MAT and BAT stand for gonadal-, subcutaneous-, mesenteric- and brown- adipose tissue.

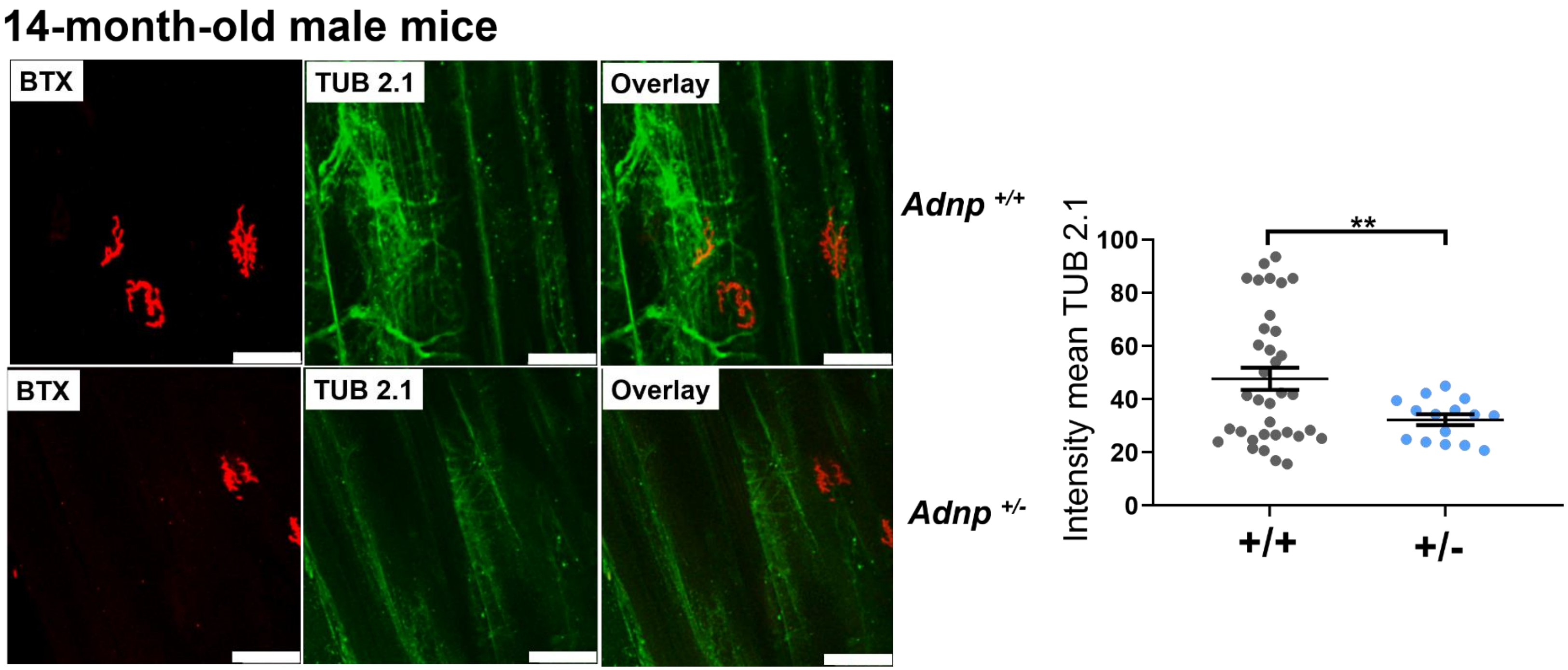

Figure S4. Representative whole-mount NMJ immunostaining of 14-month-old (*Adnp*$^{+/+}$ CB n=4; *Adnp*$^{+/-}$ CB n=2) male mice. The post-synaptic marker bungarotoxin was labeled by BTX (red) and the pre-synaptic marker Tubulin was labeled by Tub 2.1 (green). Decreased tubulin intensity was observed in *Adnp*$^{+/-}$ CB, compared with *Adnp*$^{+/+}$ CB, (**p<0.01). The images were acquired by confocal microscope at X20 magnification. Scale bar, 50 µm. The intensity mean is expressed as mean ± SEM.

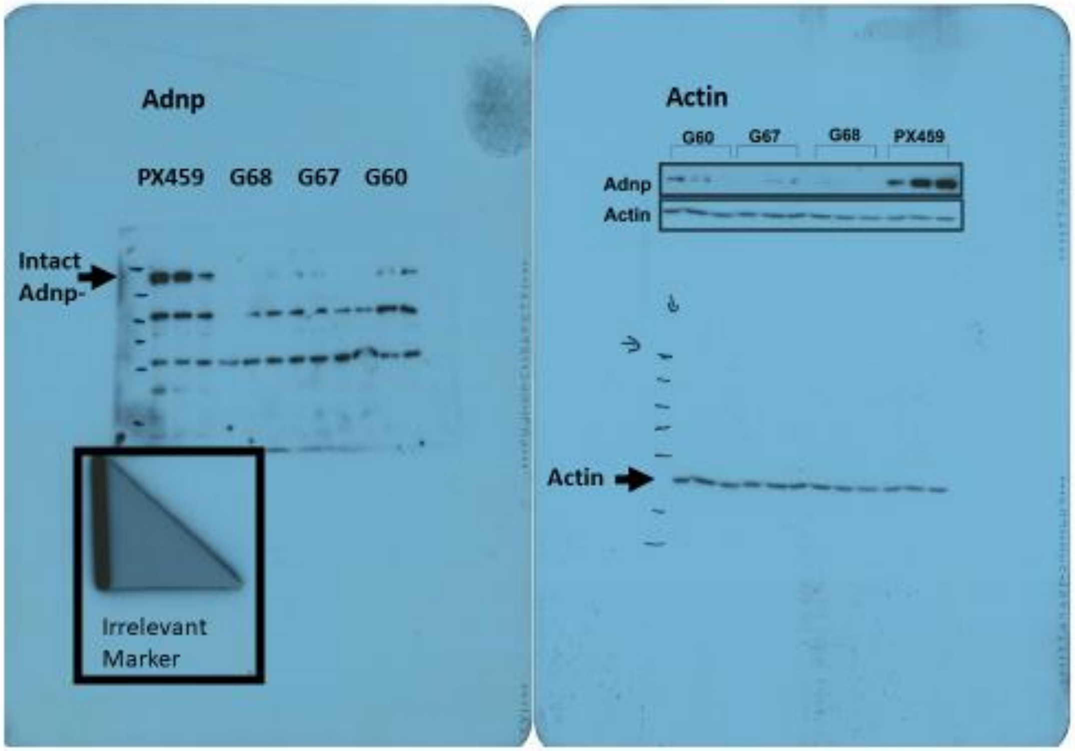

Figure S5. A successful knockdown of Adnp in culture by the CRISPR-Cas9 technology: Full Western blot control for Figure 7B. The left hand panel depicts the full Adnp Western blot results (an irrelevant marker was inserted for placement). Intact Adnp is indicated by a black arrow (experiments were performed in triplicates, as shown in the main text and in the insert – on the right side of the figure). The lower molecular weight bands identified by the Adnp antibody may represent breakdown products or non-specific antibody label. Regardless, complete knockdown of the intact Adnp is shown for the G68 sgRNA. The actin detecting antibody is highly specific (right hand panel).

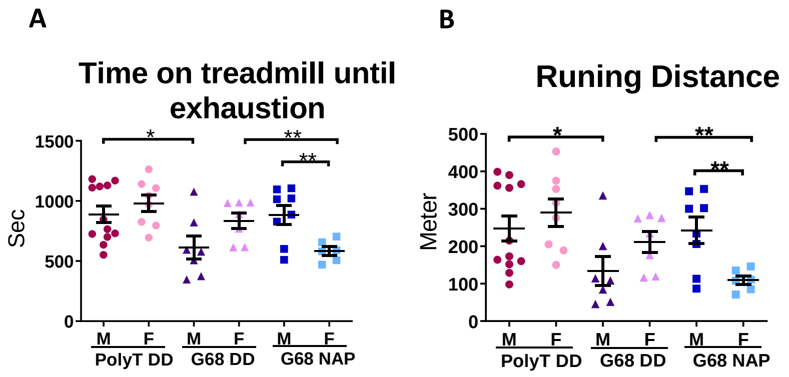

Figure S6. G68 male mice displayed a significantly reduced max ability to run on treadmill compared with the Poly T DD group. An unpaired Student's t-test revealed a significant reduction in time until exhaustion (A) and the running distance (B) in G68 male mice group compared to the control group (Poly T DD) (*p<0.05). Also, sex differences were observed in NAP treated G68 group (**p<0.01). Poly T (males n=12; females n=8), G68 DD (males n=7; females n=7) and G68 NAP groups (males n= 8; females n=7). All the results are expressed as mean ± SEM.

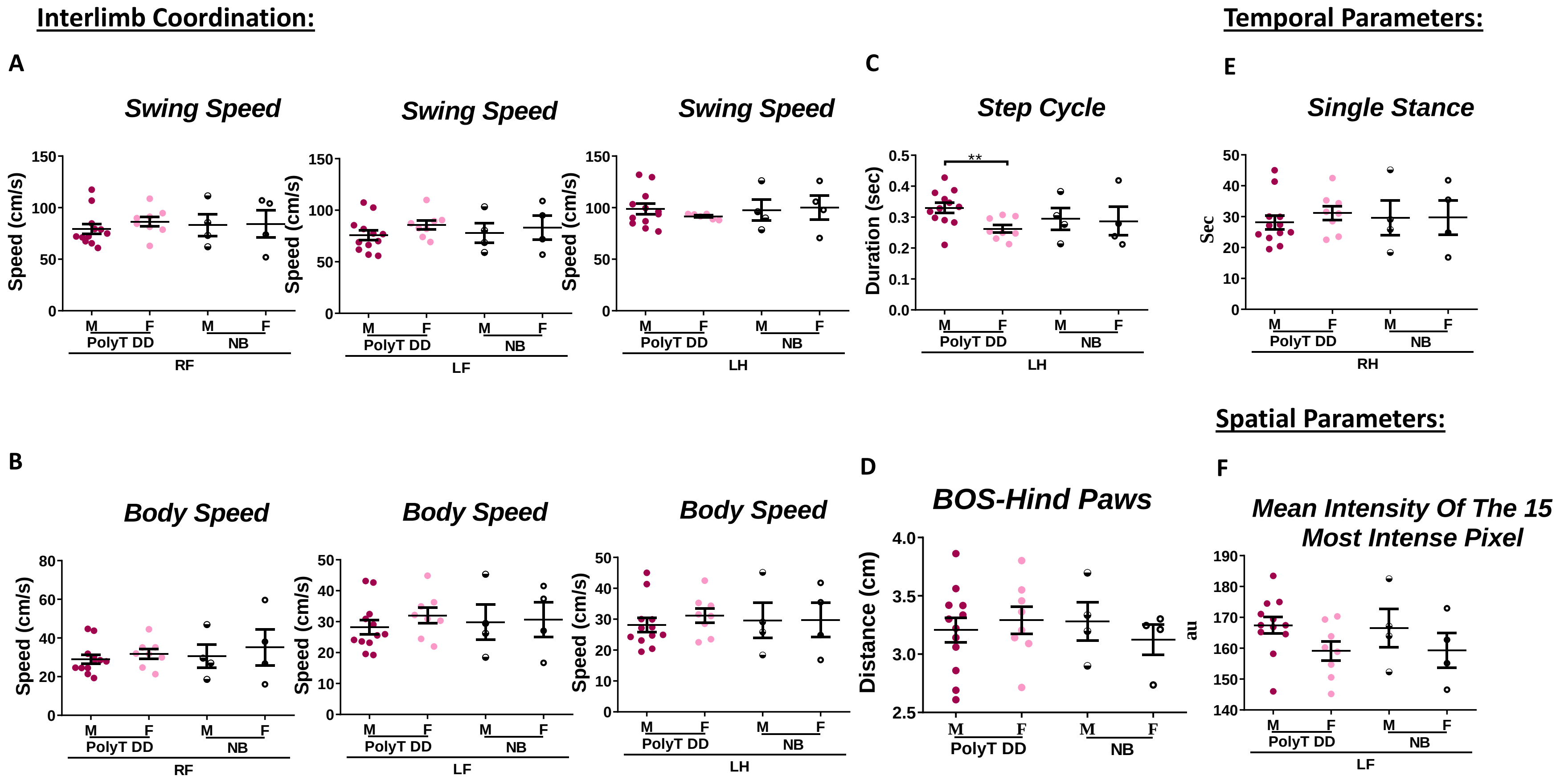

Figure S7. No significant differences were found between the Poly T DD and the neurobasal (NB) medium injected groups. Poly T (males n=12; females n=8), G68 DD (males n=7; females n=7) and G68 NAP groups (males n= 8; females n=7). All the results are expressed as mean ± SEM.
